# Long-Term Clinical Outcomes for Non-ST Elevation Acute Coronary Syndrome Patients with High-Risk Angiographic Findings Undergoing Percutaneous Coronary Intervention

**DOI:** 10.1155/2020/2139617

**Published:** 2020-05-07

**Authors:** Sida Jia, Ce Zhang, Yue Liu, Deshan Yuan, Xueyan Zhao, Runlin Gao, Yuejin Yang, Bo Xu, Zhan Gao, Jinqing Yuan

**Affiliations:** Fu Wai Hospital, National Center for Cardiovascular Diseases, Peking Union Medical College & Chinese Academy of Medical Sciences, Beijing, China

## Abstract

**Objective:**

We aim to evaluate the long-term prognosis of non-ST elevation acute coronary syndrome (NSTE-ACS) patients with high-risk coronary anatomy (HRCA).

**Background:**

Coronary disease severity is important for therapeutic decision-making and prognostication among patients presenting with NSTE-ACS. However, long-term outcome in patients undergoing percutaneous coronary intervention (PCI) with HRCA is still unknown.

**Method:**

NSTE-ACS patients undergoing PCI in Fuwai Hospital in 2013 were prospectively enrolled and subsequently divided into HRCA and low-risk coronary anatomy (LRCA) groups according to whether angiography complies with the HRCA definition. HRCA was defined as left main disease >50%, proximal LAD lesion >70%, or 2- to 3- vessel disease involving the LAD. Prognosis impact on 2-year and 5-year major adverse cardiovascular and cerebrovascular events (MACCE) is analyzed.

**Results:**

Out of 4,984 enrolled patients with NSTE-ACS, 3,752 patients belonged to the HRCA group, while 1,232 patients belonged to the LRCA group. Compared with the LRCA group, patients in the HRCA group had worse baseline characteristics including higher age, more comorbidities, and worse angiographic findings. Patients in the HRCA group had higher incidence of unplanned revascularization (2 years: 9.7% vs. 5.1%, *p* < 0.001; 5 years: 15.4% vs. 10.3%, *p* < 0.001), 2-year MACCE (13.1% vs. 8.8%, *p* < 0.001), and 5-year death/MI/revascularization/stroke (23.0% vs. 18.4%, *p* = 0.001). Kaplan–Meier survival analysis showed similar results. After adjusting for confounding factors, HRCA is independently associated with higher risk of revascularization (2 years: HR = 1.636, 95% CI: 1.225–2.186; 5 years: HR = 1.460, 95% CI: 1.186–1.798), 2-year MACCE (HR = 1.275, 95% CI = 1.019–1.596) and 5-year death/MI/revascularization/stroke (HR = 1.183, 95% CI: 1.010–1.385).

**Conclusion:**

In our large cohort of Chinese patients, HRCA is an independent risk factor for long-term unplanned revascularization and MACCE.

## 1. Introduction

According to current guidelines, for patients presenting with non-ST elevation acute coronary syndromes (NSTE-ACS), coronary disease severity plays an important role in determining the optimal treatment strategy [1, 2]. The SYNTAX score is a widely used angiographic tool to help reflect the severity of coronary artery disease and predict patient prognosis [3]. However, accurate SYNTAX score measurement requires the assistance of Angiographic Core Lab (ACL) technicians or especially trained interventional cardiologists [4]. It is reasonable to infer that if NSTE-ACS patients present to emergency department during the off-clock hours of the senior cardiologists and ACL technicians, overestimation or underestimation of the disease severity might occur. Thus, it is necessary to find a more convenient alternative to SYNTAX score, especially in the acute clinical settings of NSTE-ACS.

In an effort to better identify NSTE-ACS patients with higher disease severity and to assess their prognosis, Beigel et al. [5] evaluated 923 NSTE-ACS patients with or without high-risk coronary anatomy (HRCA), and they found that HRCA was a predictor of 30-day MACCE and 1-year mortality. HRCA was defined according to three simple angiographic criteria and was suggested to be included in the risk stratification of patients with NSTE-ACS [5]. However, the study was modest in sample size and length of follow-up, and, to the best of our knowledge, its conclusion has not been verified in larger studies.

Here, we aim to evaluate the long-term prognosis of NSTE-ACS patients with HRCA in our real-world, prospective, large-sample cohort of Chinese patients.

## 2. Materials and Methods

### 2.1. Study Population

Data from all consecutive patients from a single center (Fuwai Hospital, National Center for Cardiovascular Diseases, Beijing, China) undergoing PCI were prospectively collected. Between January 2013 and December 2013, a total of 10,724 consecutive patients were enrolled undergoing PCI. The Institutional Review Board approved the study protocol, and the patients provided written informed consent before the intervention.

**Figure 1:**
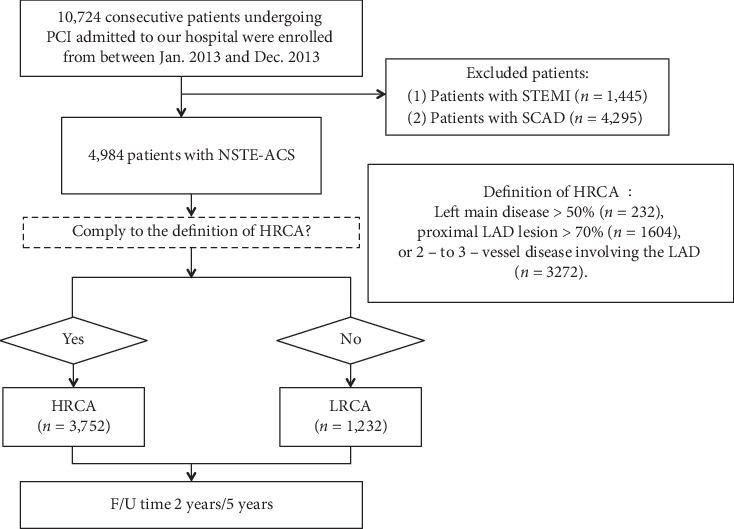
Patient flowchart. PCI = percutaneous coronary intervention; HRCA = high-risk coronary anatomy; LRCA = low-risk coronary anatomy; STEMI = ST elevation myocardial infarction; SCAD = stable coronary artery disease; NSTE-ACS = non-ST elevation acute coronary syndrome; LM = left main coronary artery; LAD = left anterior descending artery; F/U = follow-up.

Exclusion criteria included patients presenting with stable coronary artery disease (*n* = 4,295) and ST-segment elevation myocardial infarction (*n* = 1,445). Patients were subsequently stratified into HRCA or low-risk coronary anatomy (LRCA) group. HRCA was defined as one of the following: left main stenosis >50%, proximal LAD lesion >70%, and/or 2- to 3-vessel disease involving the LAD [5]. Patients not belonging to HRCA were defined as LRCA (Figure 1). Percentage stenosis of CAD lesions are based on visual assessment, determined by a team of trained physicians.

### 2.2. Procedure and Medications

The PCI strategy and stent type were left to treating physician's discretion. ACS patients (STEMI and NSTE-ACS) scheduled for PCI received 300 mg aspirin and ticagrelor (loading dose 180 mg) or clopidogrel (loading dose 300 mg or 600 mg) as soon as possible. During the procedure, unfractionated heparin (100 U/kg) was administered to all patients, and use of glycoprotein IIb/IIIa inhibitors was per operator's judgment. After the procedure, aspirin was prescribed at a dose of 100 mg daily indefinitely; clopidogrel 75 mg daily or ticagrelor 90 mg twice daily was advised for at least 1 year after PCI.

### 2.3. Patient Follow-Up

All patients were evaluated by clinic visit or by telephone interview at 1, 6, and 12 months and annually thereafter. Patients were advised to return for coronary angiography if clinically indicated by symptoms or documentation of myocardial ischemia.

### 2.4. Endpoints and Definitions

Death that could not be attributed to a noncardiac etiology was considered cardiac death. Myocardial Infarction (MI) was defined by the third universal definition of myocardial infarction [6]. Revascularization was defined as repeated revascularization for ischemic symptoms and events driven by PCI or surgery of any vessel. Stent thrombosis (ST) was defined according to the Academic Research Consortium, including definite, probable, and possible in the analysis [7]. Bleeding was quantified according to Bleeding Academic Research Consortium (BARC) definition criteria, including type 2, 3, and 5 in the analysis [8]. Major bleeding was defined as type 3 and 5 from BARC criteria. Major adverse cardiovascular and cerebrovascular events (MACCE) were defined as the occurrence of death, MI, target vessel revascularization, ST, and stroke during follow-up. Death/MI/revascularization/stroke was defined as the occurrence of death, MI, target vessel revascularization, and stroke during follow-up. All endpoints were adjudicated centrally by two independent cardiologists, and disagreement was resolved by consensus. ST and MACCE were monitored in the first 2 years of follow-up, while all other adverse events were monitored throughout the 5-year follow-up.

### 2.5. Statistical Analysis

Continuous variables are expressed as mean ± standard deviation, and categorical variables are presented as percentages. Differences in baseline characteristics and clinical outcomes between groups were assessed using the chi-square test or Fisher's exact test for categorical variables and Student's *t*-test or the Wilcoxon rank test for continuous variables, as appropriate. Survival curves were constructed using the Kaplan–Meier method, and the log-rank test was performed to compare time to clinical endpoints. Cox regression analyses were conducted to evaluate the adjusted effect of HRCA PCI on the 2-year clinical endpoints. Clinically and statistically significant covariates were all entered into the model, and results were reported as adjusted hazard ratios together with corresponding 95% confidence intervals (CI). Missing values are imputed with median for continuous variables and with mode for categorical variables. For all analyses, a 2-sided *p* value <0.05 was considered significant. Statistical analysis was performed using IBM® SPSS® v22.0.0.0 software (SPSS Inc., Chicago, IL, USA).

## 3. Results

Follow-up was complete for 4,959 patients (99.5%) at 2 years and 4,554 patients (91.4%) at 5 years. Among 4,984 patients with NSTE-ACS, 3,752 patients were stratified as HRCA, while 1,232 patients belonged to LRCA. Compared with patients in the LRCA group, patients with HRCA were higher in age, with higher proportion of diabetes, hypertension, history of stroke, and peripheral vascular disease (all *p* < 0.05). Laboratory tests results revealed that HRCA patients had higher level of creatinine, blood glucose, and high-sensitivity C-reactive protein and lower level of glomerular filtration rate and left ventricular ejection fraction (all *p* < 0.05) (Table 1). In terms of angiographic findings, patients in the HRCA group had higher preoperative and postoperative SYNTAX scores and higher proportion of total occlusion. In addition, more patients with HRCA underwent staged PCI, IVUS scan, and IABP support (all *p* < 0.05) (Table 2).

**Figure 2:**
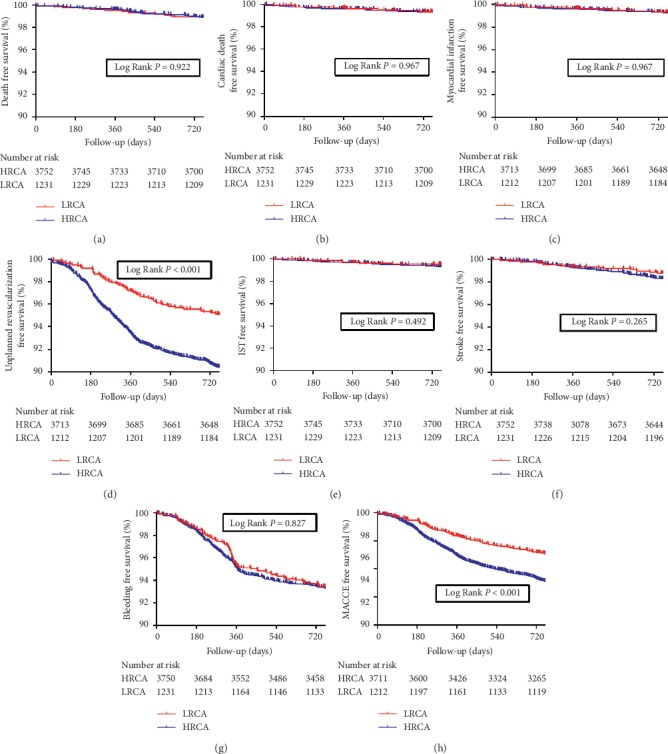
Kaplan–Meier survival analysis on 2-year clinical endpoints between HRCA and LRCA groups. (a) All-cause death; (b) cardiac death; (c) myocardial infarction; (d) unplanned revascularization; (e) in-stent thrombosis; (f) stroke; (g) bleeding; (h) MACCE. MACCE = major adverse cardiac and cerebrovascular events.

**Figure 3:**
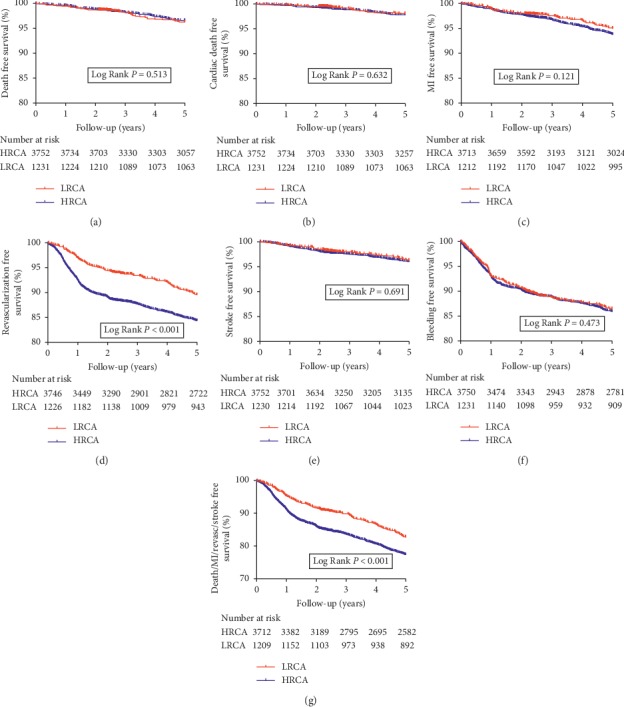
Kaplan–Meier survival analysis on 5-year clinical endpoints between HRCA and LRCA groups. (a) All-cause death; (b) cardiac death; (c) myocardial infarction; (d) unplanned revascularization; (e) stroke; (f) bleeding; (g) death/MI/revascularization/stroke.

At 2 years, follow-up results revealed that patients with HRCA had a significantly higher incidence of 2-year unplanned revascularization (9.7% vs. 5.1%, *p* < 0.001) and MACCE (13.1% vs. 8.8%, *p* < 0.001) compared with patients with LRCA. Meanwhile, no significant difference was found in the incidence of 2-year all-cause death, cardiac death, myocardial infarction, in-stent thrombosis, stroke, and bleeding (all *p* > 0.05) (Table 3). Similarly, at 5 years, patients in the HRCA group had significantly higher incidence of 5-year unplanned revascularization (15.4% vs. 10.3%, *p* < 0.001) and death/MI/revascularization/stroke (23.0% vs. 18.4%, *p* < 0.001). No significant difference was found in other clinical endpoints (all *p* > 0.05) (Supplementary Table 1). Kaplan–Meier survival analysis revealed similar results Figures 2 and 3.

After adjusting for confounding factors between HRCA and LRCA groups by multivariate Cox regression analysis, HRCA remained an independent risk factor for unplanned revascularization (2-year: HR = 1.636, 95% CI: 1.225–2.186; 5-year: HR = 1.460, 95% CI: 1.186–1.798) and 2-year MACCE (HR = 1.275, 95% CI: 1.019–1.596) and 5-year death/MI/revascularization/stroke (HR = 1.183, 95% CI: 1.010–1.385). Adjusted variables included age, diabetes, hypertension, previous stroke, peripheral vascular disease, preprocedural creatinine, preprocedural glomerular filtration rate, left ventricular ejection fraction, preprocedural blood glucose, hsCRP, *β*-blocker usage, preprocedural SYNTAX score, IVUS usage, IABP usage, PTCA only, BMS implantation, second generation DES implantation, biodegradable polymer DES implantation, and other types of stent implantation (Table 4, Supplementary Table 2).

Subgroup analysis revealed that the impact of HRCA on 2-year unplanned revascularization (Table 5) and MACCE (Table 6) were consistent across all subgroups, including age, gender, diabetes, LVEF, SYNTAX score, baseline GFR, stent types, and IVUS usage (all *p* for interaction >0.05). Meanwhile, the impact of HRCA on 5-year unplanned revascularization and death/MI/revascularization/stroke is also consistent across subgroups except for baseline GFR, where a significant interaction was found on 5-year unplanned revascularization (*p* for interaction = 0.016) (Supplementary Tables 3 and 4).

## 4. Discussion

With the improvement in early diagnosis of ST elevation myocardial infarction (STEMI) in China, there is an increase of patients with relatively moderate types of ACS, the NSTE-ACS [9]. In search of a more convenient way to better risk-stratify NSTE-ACS patients, we compared the long-term prognosis in patients with or without HRCA, a previously reported tool based on three simple angiographic criteria. The major findings of our study include: (1) Patients with HRCA tend to have worse baseline conditions and more comorbidities. (2) NSTE-ACS patients with HRCA had a significantly higher rate of 2-year and 5-year unplanned revascularization, 2-year MACCE, and 5-year death/MI/revascularization/stroke. (3) HRCA is an independent risk factor for a 2-year and 5-year unplanned revascularization, 2-year MACCE, and 5-year death/MI/revascularization/stroke in patients with NSTE-ACS.

Over the past decade, a number of risk-scoring tools have been developed to predict the prognosis of NSTE-ACS patients. The TIMI risk score for unstable angina/non-ST elevation MI, based on 7 predictor variables including clinical presentation and risk factors, is a simple prognostication scheme categorizing patients' risk of death [10]. In addition, both the GRACE risk score and the simplified GRACE risk score 2.0 are well validated tools for estimating in-hospital and long-term risk in ACS patients [11, 12]. However, these scoring tools did not include angiographic disease severity as a factor in their predictive models and have only modest value in predicting the angiographic severity [13, 15].

Lesions located in the LM and proximal LAD or LAD lesions in multivessel CAD constitute a high-risk angiographic profile, mainly due to the large myocardial perfusion territory these vessels supply [16]. The high-risk nature of HRCA definition is further supported by the coronary segment weighting factors in SYNTAX score calculation, where lesions in LAD (especially proximal LAD) and LM have the highest weighting factor in the whole coronary artery system irrespective of coronary dominance pattern [17]. According to the study by Beigel et al., adding HRCA to GRACE score significantly increases net reclassification index for 30-day MACE and mortality but not for 1-year mortality [5]. Similarly, no significant difference was found in long-term incidence of all-cause and cardiac mortality between HRCA and LRCA groups in our study. However, we found HRCA was independently associated with significantly increased risk of 2-year MACCE and 5-year death/MI/revascularization/stroke, which is mainly driven by higher risk of unplanned revascularization. We have proposed two possible mechanisms for our results: (1) HRCA involves significant stenosis in LM and/or LAD. In-stent restenosis or newly developed de-novo lesions in LM or LAD might have a greater impact on left ventricular myocardial perfusion than in other coronary artery vessels, causing a higher rate of ischemia-driven target lesion revascularization in the long run. (2) Compared with patients with LRCA, the baseline conditions are generally worse in the HRCA group. Despite our effort to adjust for confounding factors by applying multivariate cox regression model, unknown confounding factors still exist.

It is worth noticing that the all-cause mortality rate in our study was around 3-4% at 5-year follow-up, which is significantly lower than similar studies on NSTEMI patients. In a large observational cohort study on 11,737 NSTEMI patients, mortality rate was at 22.5% and 25.9% in two study groups in a median follow-up period of 4.1 years. [18] Possible explanations include the following: (1) Our study focused on NSTE-ACS patients instead of only NSTEMI. Most of the patients presented as lower risk unstable angina (around 90%) instead of NSTEMI. (2) Although SYNTAX score is a well-established predictor for long-term mortality, it is not parallel to the definition of HRCA. The average SYNTAX score in the HRCA group was generally low, indicating lower complexity of CAD. Taken together, the lower-risk clinical presentation and lower complexity of CAD both contributed to the low mortality rate than NSTEMI studies.

There are some inherent limitations in our study. First, whether the lesion intervened was a culprit lesion causing NSTE-ACS was unknown. Second, due to the observational nature of our study, unknown confounding factors still exist. Third, procedural details that might influence clinical outcomes are missing, including post-dilatation and stenting technique for LM bifurcation lesions. Fourth, due to the low sample capacity in the low baseline GFR subgroup, subgroup analysis result is likely underpowered to confirm real-world interactions despite statistical significance. Last but not the least, our study started in 2013 when newer antiplatelet agents and newer generation stents were not so widely used. This may limit the generalizability of our finding to modern ACS settings in the US and Europe.

## 5. Conclusion

In our large cohort of Chinese patients, HRCA is an independent risk factor for long-term unplanned revascularization and MACCE. HRCA might be a convenient tool to assess coronary disease severity and predict long-term revascularization events for NSTE-ACS patients.

## Figures and Tables

**Table 1:** Baseline patient characteristics.

	HRCA (*n* = 3752)	LRCA (*n* = 1232)	*p* value
Age	59.49 ± 10.04	57.87 ± 10.15	**<0.001**
Female, *n*(%)	962 (25.6)	320 (26.0)	0.816
Body mass index, kg/m^2^	25.84 ± 3.18	25.88 ± 3.26	0.680
Risk factors and history, *n* (%)			
Smoker	2152 (57.4)	691 (56.1)	0.435
Diabetes	1173 (31.3)	299 (24.3)	**<0.001**
Hypertension	2508 (66.8)	780 (63.3)	**0.023**
Hyperlipidemia	2533 (67.5)	825 (67.0)	0.723
Prior myocardial infarction	586 (15.6)	177 (14.4)	0.290
Prior stroke	461 (12.3)	103 (8.4)	**<0.001**
Peripheral vascular disease	105 (2.8)	19 (1.5)	**0.014**
Family history of CAD	924 (24.6)	312 (25.3)	0.633
Laboratory tests			
Leukocyte, ×10^9^/L	6.83 ± 1.77	6.81 ± 1.77	0.700
Platelet, ×10^9^/L	203.44 ± 53.52	201.92 ± 48.93	0.379
Hemoglobin, g/L	140.44 ± 15.96	141.40 ± 16.39	0.072
Creatinine, umol/L	75.55 ± 16.03	73.86 ± 16.20	**0.001**
GFR, ml/min/1.73 m^2^	90.28 ± 15.11	92.87 ± 14.69	**<0.001**
LVEF, %	63.56 ± 6.69	64.07 ± 6.48	**0.023**
Glucose, mg/dL	6.17 ± 2.04	5.88 ± 1.70	**<0.001**
CK-MB, IU/L	11.83 ± 12.91	11.96 ± 10.73	0.742
BUN, mmol/L	5.70 ± 1.68	5.61 ± 1.65	0.102
hsCRP	3.18 ± 3.68	2.76 ± 3.41	**<0.001**
Clinical presentation			
NSTEMI	365 (9.7)	110 (8.9)	0.407
UA	3387 (90.3)	1122 (91.1)	0.407
Medication at discharge, *n* (%)			
Aspirin	3708 (98.8)	1215 (98.6)	0.566
Clopidogrel	3741 (99.7)	1230 (99.8)	0.747
Ticagrelor	10 (0.3)	2 (0.2)	0.517
*β*-Blockers	3356 (89.4)	1053 (85.5)	**<0.001**
Calcium channel blockers	2163 (57.6)	682 (55.4)	0.158
Nitrates	3700 (98.6)	1199 (97.3)	**0.002**
Statins	3609 (96.2)	1182 (95.9)	0.696

Values are mean ± SD or *n* (%). HRCA = high-risk coronary anatomy; CAD = coronary artery disease; GFR = glomerular filtration rate; LVEF = left ventricular ejection fraction; CK-MB = creatine kinase-muscle/brain; BUN = blood urea nitrogen; hsCRP = high-sensitivity C-reactive protein; STEMI = ST elevation myocardial infarction; NSTEMI = non-ST elevation myocardial infarction; UA = unstable angina.

**Table 2:** Coronary angiographic findings and percutaneous interventional therapies.

	HRCA (*n* = 3752)	LRCA (*n* = 1232)	*p* value
SYNTAX score			
Preprocedure	12.52 ± 8.07	6.97 ± 5.52	<0.001
Postprocedure	3.73 ± 5.83	1.47 ± 3.50	<0.001
Number of diseased vessels			
One	465 (12.4)	809 (65.7)	<0.001
Two	1341 (35.7)	313 (25.4)	<0.001
Three	1937 (51.6)	0 (0)	<0.001
Left main disease, %	369 (9.8)	14 (1.1)	<0.001
Total occlusion, %	660 (17.6)	154 (12.5)	<0.001
Puncture site, %			
Femoral artery	57 (1.5)	22 (1.8)	0.125
Radial artery	3397 (90.5)	1133 (92.0)	
Other approaches	298 (7.9)	77 (6.3)	
Staged PCI, %	380 (10.1)	37 (3.0)	<0.001
IVUS usage, %	247 (6.6)	39 (3.2)	<0.001
IABP usage, %	42 (1.1)	4 (0.3)	0.011
Successful PCI, %	3687 (98.3)	1210 (98.2)	0.901
PTCA only, %	718 (19.1)	145 (11.8)	<0.001
Stent type			
BMS %	22 (0.6)	3 (0.2)	0.139
DES, %			
1G-DES	679 (18.1)	214 (17.4)	0.564
2G-DES	1609 (42.9)	569 (46.2)	0.043
BP-DES	525 (14.0)	207 (16.8)	0.016
Others	47 (1.3)	26 (2.1)	0.030
Blended multiple DESs	12 (0.3)	9 (0.7)	0.053

Values are mean ± SD or *n* (%). RCA = high-risk coronary anatomy; LRCA = low-risk coronary anatomy; SYNTAX = SYNergy between percutaneous coronary intervention with TAXus and cardiac surgery; PCI = percutaneous coronary intervention; IVUS = intravascular ultrasound; IABP = Intra-aortic balloon pump; PTCA = percutaneous transluminal coronary angioplasty; BMS = bare metal stent; DES = drug eluting stent; 1G = first generation; 2G = second generation; BP = biodegradable polymer.

**Table 3:** Two-year clinical outcomes.

	HRCA (*n* = 3752)	LRCA (*n* = 1232)	*p* value
All-cause death	41 (1.1)	14 (1.1)	0.899
Cardiac death	23 (0.6)	6 (0.5)	0.614
Myocardial infarction	64 (1.7)	28 (2.3)	0.200
Unplanned revascularization	363 (9.7)	63 (5.1)	<0.001
In-stent thrombosis	33 (0.9)	8 (0.6)	0.438
Stroke	63 (1.7)	15 (1.2)	0.257
Bleeding	252 (6.7)	80 (6.5)	0.785
MACCE	493 (13.1)	108 (8.8)	<0.001

Values are *n* (%). HRCA = high-risk coronary anatomy; LRCA = low-risk coronary anatomy; MACCE = major adverse cardiac and cerebrovascular events.

**Table 4:** Multivariable cox regression analysis of HRCA on clinical outcomes.

	HRCA	*p*
	Hazard ratio (95% confidence interval)	
All-cause death	0.885 (0.465–1.682)	0.708
Cardiac death	1.164 (0.439–3.084)	0.760
Myocardial infarction	0.623 (0.383–1.011)	0.055
Revascularization	1.636 (1.225–2.186)	**0.001**
In-stent thrombosis	1.185 (0.524–2.682)	0.683
Stroke	1.194 (0.649–2.198)	0.568
Bleeding	1.133 (0.860–1.492)	0.375
MACCE	1.275 (1.019–1.596)	**0.034**

HRCA = high risk coronary anatomy; LRCA = low-risk coronary anatomy; MACCE = major adverse cardiac and cerebrovascular events. Adjusted variables: age, diabetes, hypertension, previous stroke, peripheral vascular disease, preprocedural creatinine, preprocedural glomerular filtration rate, left ventricular ejection fraction, preprocedural blood glucose, hsCRP, *β*-blocker usage, preprocedural SYNTAX score, IVUS usage, IABP usage, PTCA only, BMS implantation, second generation DES implantation, biodegradable polymer DES implantation, and other types of stent implantation.

**Table 5:** Subgroup analysis on MACCE between HRCA and LRCA.

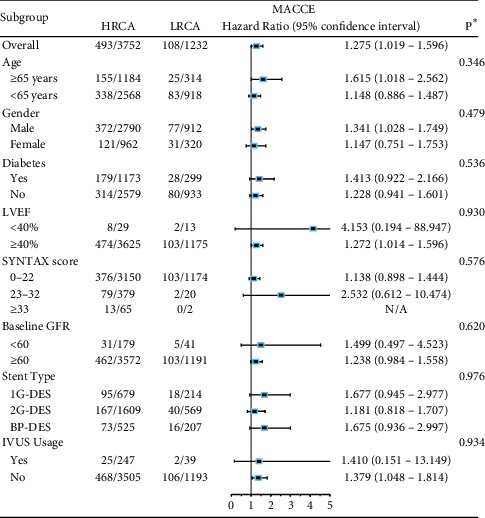

^*∗*^ *p* value for interaction in each subgroup analysis. HRCA = high risk coronary anatomy; LRCA = low-risk coronary anatomy; MACCE = major adverse cardiac and cerebrovascular events. Adjusted variables: age, diabetes, hypertension, previous stroke, peripheral vascular disease, preprocedural creatinine, preprocedural glomerular filtration rate, left ventricular ejection fraction, preprocedural blood glucose, hsCRP, *β*-blocker usage, preprocedural SYNTAX score, staged PCI, IVUS usage, IABP usage, stent type, and HRCA.

**Table 6:** Subgroup analysis on unplanned revascularization between HRCA and LRCA.

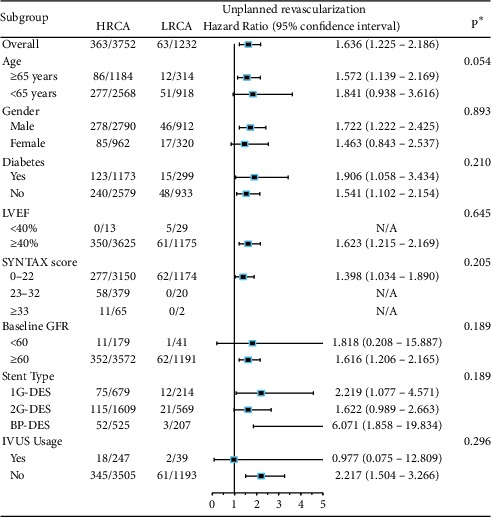

^*∗*^ *p* value for interaction in each subgroup analysis. HRCA = high risk coronary anatomy; LRCA = low-risk coronary anatomy; MACCE = major adverse cardiac and cerebrovascular events. Adjusted variables: age, diabetes, hypertension, previous stroke, peripheral vascular disease, preprocedural creatinine, preprocedural glomerular filtration rate, left ventricular ejection fraction, preprocedural blood glucose, hsCRP, *β*-blocker usage, preprocedural SYNTAX score, staged PCI, IVUS usage, IABP usage, stent type, and HRCA.

## Data Availability

The clinical data used to support the findings of this study are available from the corresponding author upon request.

## Supplementary Materials

Supplementary Materials Supplementary Materials. Supplementary Table 1: five-year clinical outcome. Supplementary Table 2: multivariable cox regression analysis of HRCA on 5-year clinical outcomes. Supplementary Table 3: subgroup analysis on death/MI/revascularization/stroke (5-year results). Supplementary Table 4: subgroup analysis on unplanned revascularization between HRCA and LRCA (5-year results).
