# Supplementary material for: Long-Term Clinical Outcomes for Non-ST Elevation Acute Coronary Syndrome Patients with High-Risk Angiographic Findings Undergoing Percutaneous Coronary Intervention
Source: J Interv Cardiol. 2020 May 7;2020:2139617. doi: 10.1155/2020/2139617 (PMC7229566; doi:10.1155/2020/2139617)
Supplement: Supplementary Materials — Supplementary Table 1: five-year clinical outcome. Supplementary Table 2: multivariable cox regression analysis of HRCA on 5-year clinical outcomes. Supplementary Table 3: subgroup analysis on death/MI/revascularization/stroke (5-year results). Supplementary Table 4: subgroup analysis on unplanned revascularization between HRCA and LRCA (5-year results). [file 2139617.f1.pdf]

Supplementary Table 1. Five-year clinical outcome

	HRCA (n=3752)	LRCA (n=1232)	P value
All-cause Death	126 (3.4)	47 (3.8)	0.447
Cardiac Death	79 (2.1)	24 (1.9)	0.736
Myocardial Infarction	262 (7.0)	78 (6.3)	0.431
Unplanned Revascularization	576 (15.4)	127 (10.3)	**<0.001**
Stroke	143 (3.8)	44 (3.6)	0.701
Bleeding	525 (14.0)	161 (13.1)	0.414
Death/MI/revascularization/stroke	863 (23.0)	227 (18.4)	**0.001**

Values are n (%).

HRCA = High Risk Coronary Anatomy; LRCA = Low Risk Coronary Anatomy;

Supplementary Table 2. Multivariable Cox Regression Analysis of HRCA on 5-year clinical outcomes

	HRCA	
	Hazard Ratio (95% Confidence Interval)	P value
All-cause Death	0.816 (0.570 - 1.168)	0.266
Cardiac Death	1.005 (0.620 - 1.631)	0.983
Myocardial Infarction	1.068 (0.812 - 1.405)	0.637
Unplanned Revascularization	1.460 (1.186 - 1.798)	<0.001
Stroke	0.911 (0.631 - 1.313)	0.616
Bleeding	1.208 (0.994 - 1.467)	0.057
Death/MI/revascularization/stroke	1.183 (1.010 - 1.385)	0.037

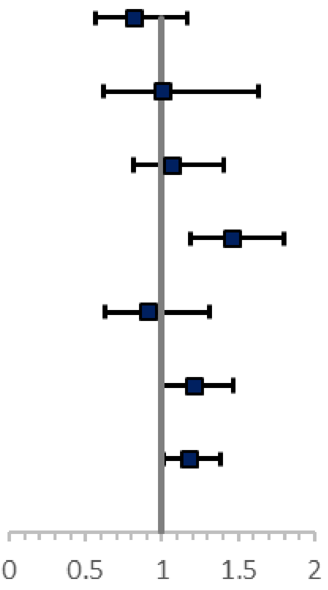

HRCA = High-risk Coronary Anatomy; LRCA = Low Risk Coronary Anatomy; MACCE = Major Adverse Cardiac and Cerebrovascular Events.

Adjusted variables: age, diabetes, hypertension, previous stroke, peripheral vascular disease, preprocedural creatinine, preprocedural glomerular filtration rate, left ventricular ejection fraction, preprocedural blood glucose, hsCRP, β-blocker usage, preprocedural SYNTAX score, IVUS usage, IABP usage, PTCA only, BMS implantation, second generation DES implantation, biodegradable polymer DES implantation, other types of stent implantation

Supplementary Table 3. Subgroup analysis on death/MI/revascularization/stroke (5-year results)

Subgroup	Death/MI/revascularization/stroke			
	HRCA	LRCA	Hazard Ratio (95% Confidence Interval)	P*
Overall	863/3752	227/1232	1.183 (1.010 - 1.385)	
Age				0.522
⩾65 years	303/1184	63/314	1.333 (0.997 - 1.780)	
<65 years	560/2568	164/918	1.097 (0.908 - 1.326)	
Gender				0.355
Male	647/2790	161/912	1.252 (1.039 - 1.509)	
Female	216/962	66/320	1.023 (0.759 - 1.379)	
Diabetes				0.498
Yes	302/1173	56/299	1.304 (0.962 - 1.767)	
No	314/2579	80/933	1.135 (0.942 - 1.367)	
LVEF				0.537
<40%	11/29	3/13	6.669 (0.427 - 104.251)	
⩾40%	836/3625	217/1175	1.175 (1.002 - 1.377)	
SYNTAX score				0.412
0-22	679/3150	215/1174	1.091 (0.923 - 1.290)	
23-32	109/379	6/20	1.156 (0.500 - 2.673)	
⩾33	18/65	0/2	N/A	
Baseline GFR				0.483
<60	52/179	12/41	1.125 (0.557 - 2.273)	
≥60	811/3572	215/1191	1.164 (0.990 - 1.370)	
Stent Type				0.928
1G-DES	172/679	38/214	1.410 (0.956 - 2.079)	
2G-DES	314/1609	91/569	1.098 (0.854 - 1.412)	
BP-DES	138/525	36/207	1.551 (1.048 - 2.297)	
IVUS Usage				0.668
Yes	51/247	5/39	1.548 (0.575 - 4.169)	
No	812/3505	222/1193	1.167 (0.994 - 1.370)	

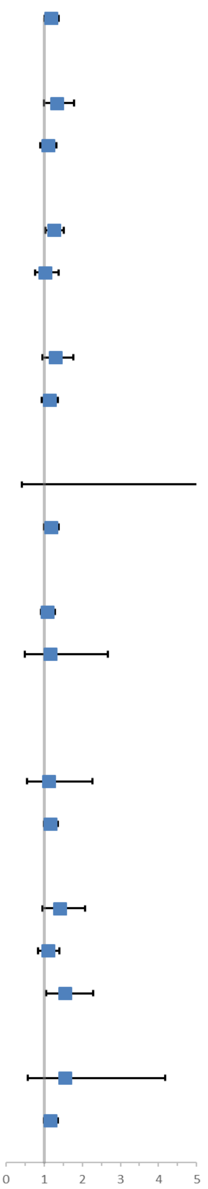

*P value for interaction in each subgroup analysis

HRCA = High-risk Coronary Anatomy; LRCA = Low Risk Coronary Anatomy; MACCE = Major Adverse Cardiac and Cerebrovascular Events.

Adjusted variables: age, diabetes, hypertension, previous stroke, peripheral vascular disease, preprocedural creatinine, preprocedural glomerular filtration rate, left ventricular ejection fraction, preprocedural blood glucose, hsCRP, β-blocker usage, preprocedural SYNTAX score, IVUS usage, IABP usage, PTCA only, BMS implantation, second generation DES implantation, biodegradable polymer DES implantation, other types of stent implantation

Supplementary Table 4 . Subgroup analysis on unplanned revascularization between HRCA and LRCA (5-year results)

Subgroup	Unplanned revascularization			
	HRCA	LRCA	Hazard Ratio (95% Confidence Interval)	P*
Overall	576/3752	127/1232	1.460 (1.186 - 1.798)	
Age				0.403
⩾65 years	157/1184	25/314	0.925 (0.563 - 1.519)	
<65 years	423/2568	106/918	1.470 (1.161 - 1.861)	
Gender				0.078
Male	443/2790	100/912	1.402 (1.095 - 1.795)	
Female	137/962	31/320	1.080 (0.674 - 1.730)	
Diabetes				0.717
Yes	192/1173	32/299	1.346 (0.883 - 2.050)	
No	388/2579	99/933	1.356 (1.060 - 1.735)	
LVEF				0.119
<40%	1/13	5/29	N/A	
⩾40%	564/3625	125/1175	1.340 (1.088 - 1.652)	
SYNTAX score				0.960
0-22	458/3150	126/1174	1.287 (1.032 - 1.605)	
23-32	75/379	2/20	3.322 (0.541 - 20.387)	
⩾33	13/65	0/2	N/A	
Baseline GFR				**0.016**
<60	25/179	5/41	2.023 (0.257 - 15.944)	
≥60	555/3572	126/1191	1.368 (1.105 - 1.695)	
Stent Type				0.193
1G-DES	116/679	23/214	1.054 (0.612 - 1.814)	
2G-DES	201/1609	51/569	1.509 (1.067 - 2.132)	
BP-DES	92/525	13/207	2.391 (1.204 - 4.749)	
IVUS Usage				0.765
Yes	30/247	5/39	0.962 (0.177 - 5.239)	
No	550/3505	126/1193	1.351 (1.091 - 1.672)	

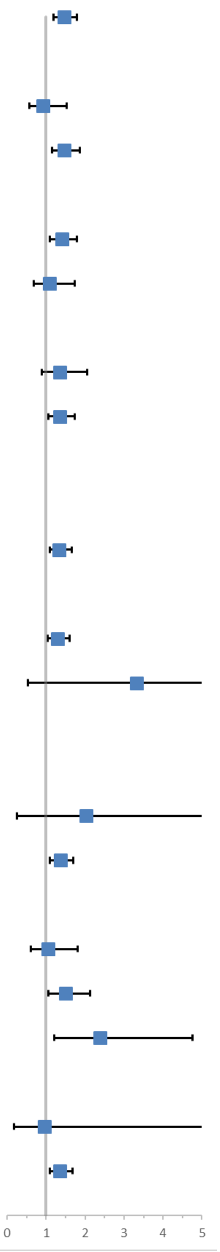

*P value for interaction in each subgroup analysis

HRCA = High-risk Coronary Anatomy; LRCA = Low Risk Coronary Anatomy; MACCE = Major Adverse Cardiac and Cerebrovascular Events.

Adjusted variables: age, diabetes, hypertension, previous stroke, peripheral vascular disease, preprocedural creatinine, preprocedural glomerular filtration rate, left ventricular ejection fraction, preprocedural blood glucose, hsCRP, β-blocker usage, preprocedural SYNTAX score, IVUS usage, IABP usage, PTCA only, BMS implantation, second generation DES implantation, biodegradable polymer DES implantation, other types of stent implantation
